# Supplementary figures and images for: TSG-6 in extracellular vesicles from canine mesenchymal stem/stromal is a major factor in relieving DSS-induced colitis
Source: PLoS One. 2020 Feb 10;15(2):e0220756. doi: 10.1371/journal.pone.0220756 (PMC7010233; doi:10.1371/journal.pone.0220756)

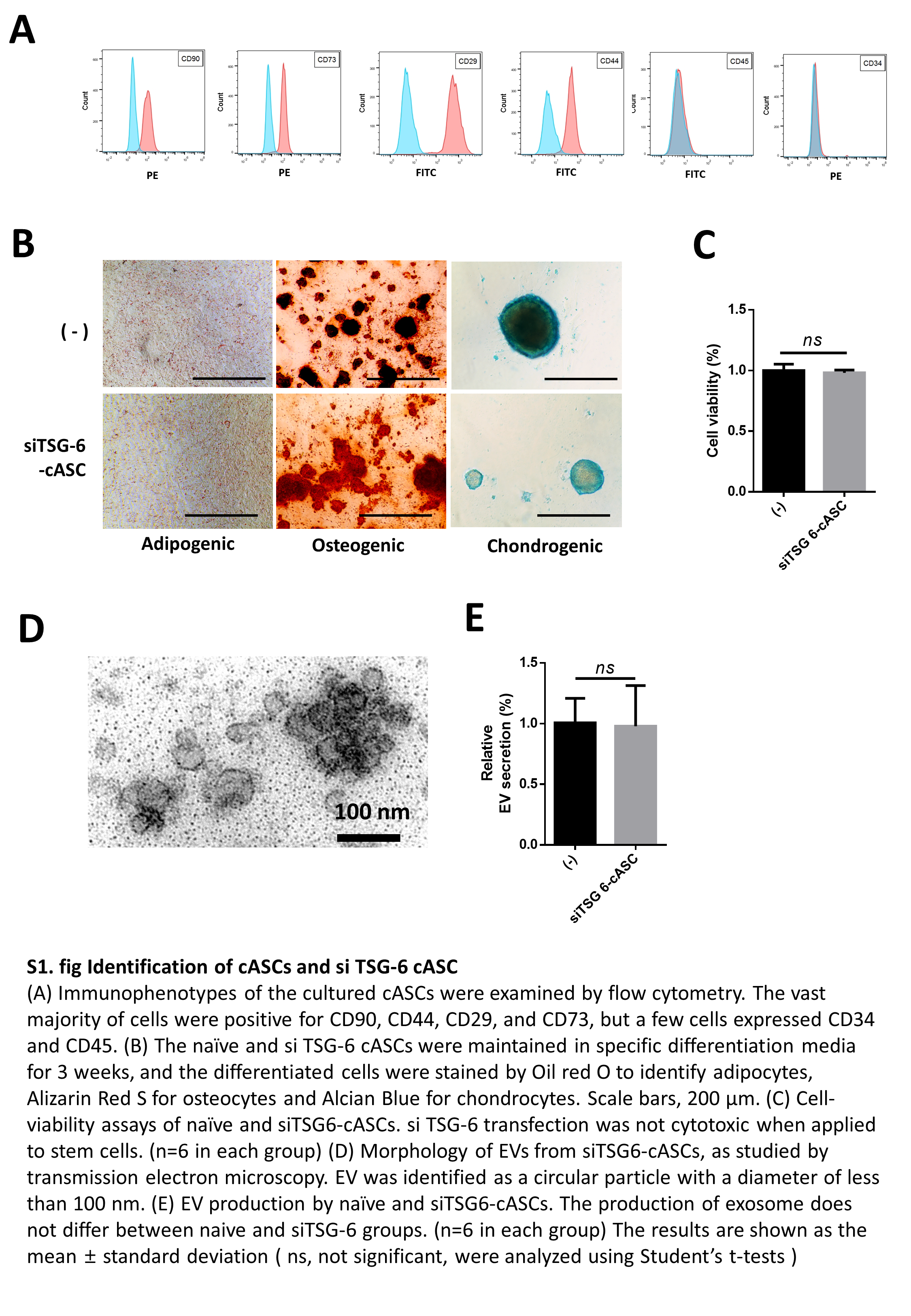

Supplement: S1 Fig — (A) Immunophenotypes of the cultured cASCs were examined by flow cytometry. The vast majority of cells were positive for CD90, CD44, CD29, and CD73, but a few cells expressed CD34 and CD45. (B) The naïve and si TSG-6 cASCs were maintained in specific differentiation media for 3 weeks, and the differentiated cells were stained by Oil red O to identify adipocytes, Alizarin Red S for osteocytes and Alcian Blue for chondrocytes. Scale bars, 200 μm. (C) Cell-viability assays of naïve and siTSG6-cASCs. si TSG-6 transfection was not cytotoxic when applied to stem cells. (n = 6 in each group) (D) Morphology of EVs from siTSG6-cASCs, as studied by transmission electron microscopy. EV was identified as a circular particle with a diameter of less than 100 nm. (E) EV production by naïve and siTSG6-cASCs. The production of exosome does not differ between naive and siTSG-6 groups. (n = 6 in each group) The results are shown as the mean ± standard deviation (ns, not significant, were analyzed using Student’s t-tests) (TIF) [file pone.0220756.s001.TIF]

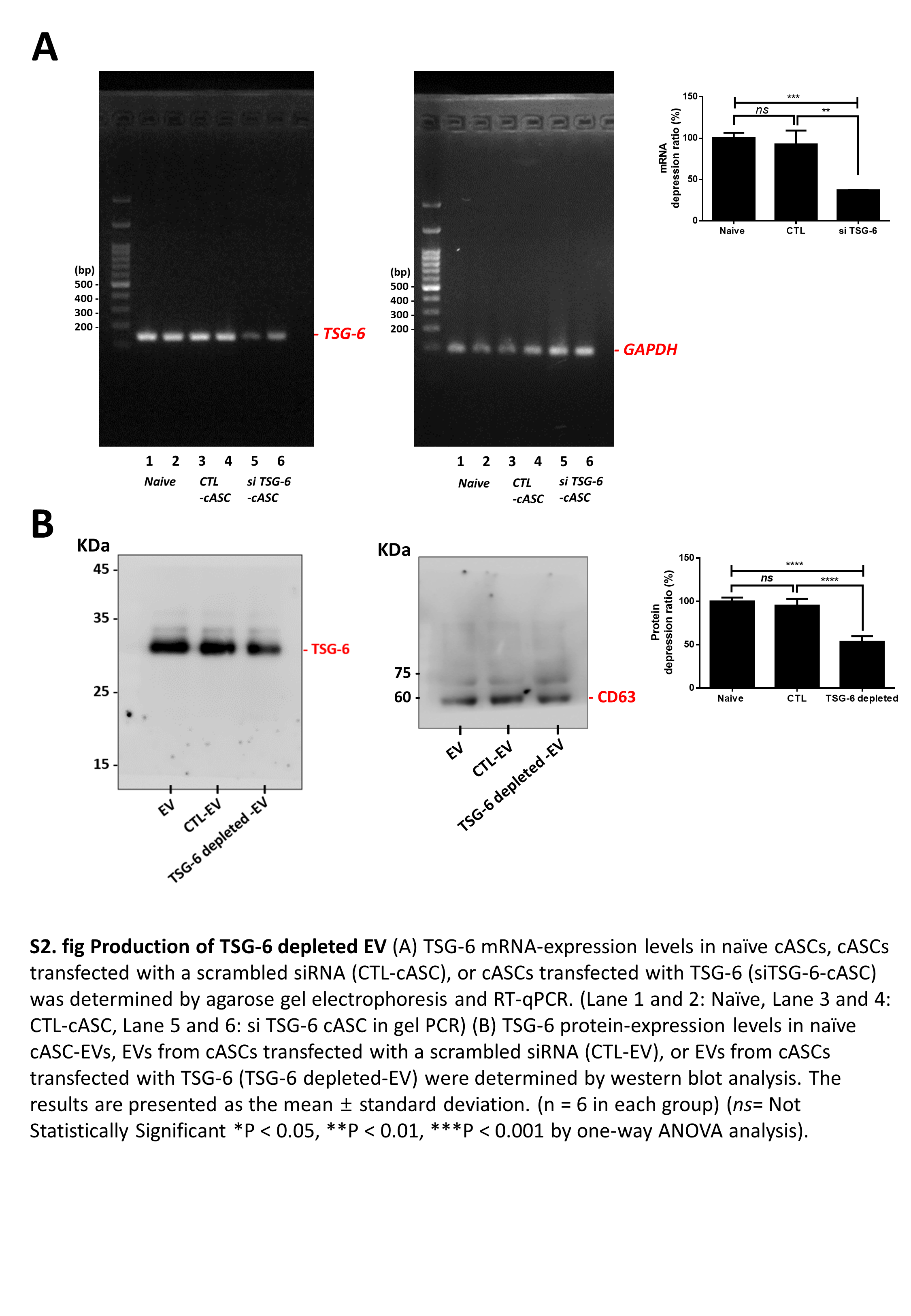

Supplement: S2 Fig — (A) TSG-6 mRNA-expression levels in naïve cASCs, cASCs transfected with a scrambled siRNA (CTL-cASC), or cASCs transfected with TSG-6 (siTSG-6-cASC) was determined by agarose gel electrophoresis and RT-qPCR. (Lane 1 and 2: Naïve, Lane 3 and 4: CTL-cASC, Lane 5 and 6: si TSG-6 cASC in gel PCR) (B) TSG-6 protein-expression levels in naïve cASC-EVs, EVs from cASCs transfected with a scrambled siRNA (CTL-EV), or EVs from cASCs transfected with TSG-6 (TSG-6 depleted-EV) were determined by western blot analysis. The results are presented as the mean ± standard deviation. (n = 6 in each group) (ns = Not Statistically Significant *P < 0.05, **P < 0.01, ***P < 0.001 by one-way ANOVA analysis). (TIF) [file pone.0220756.s002.TIF]

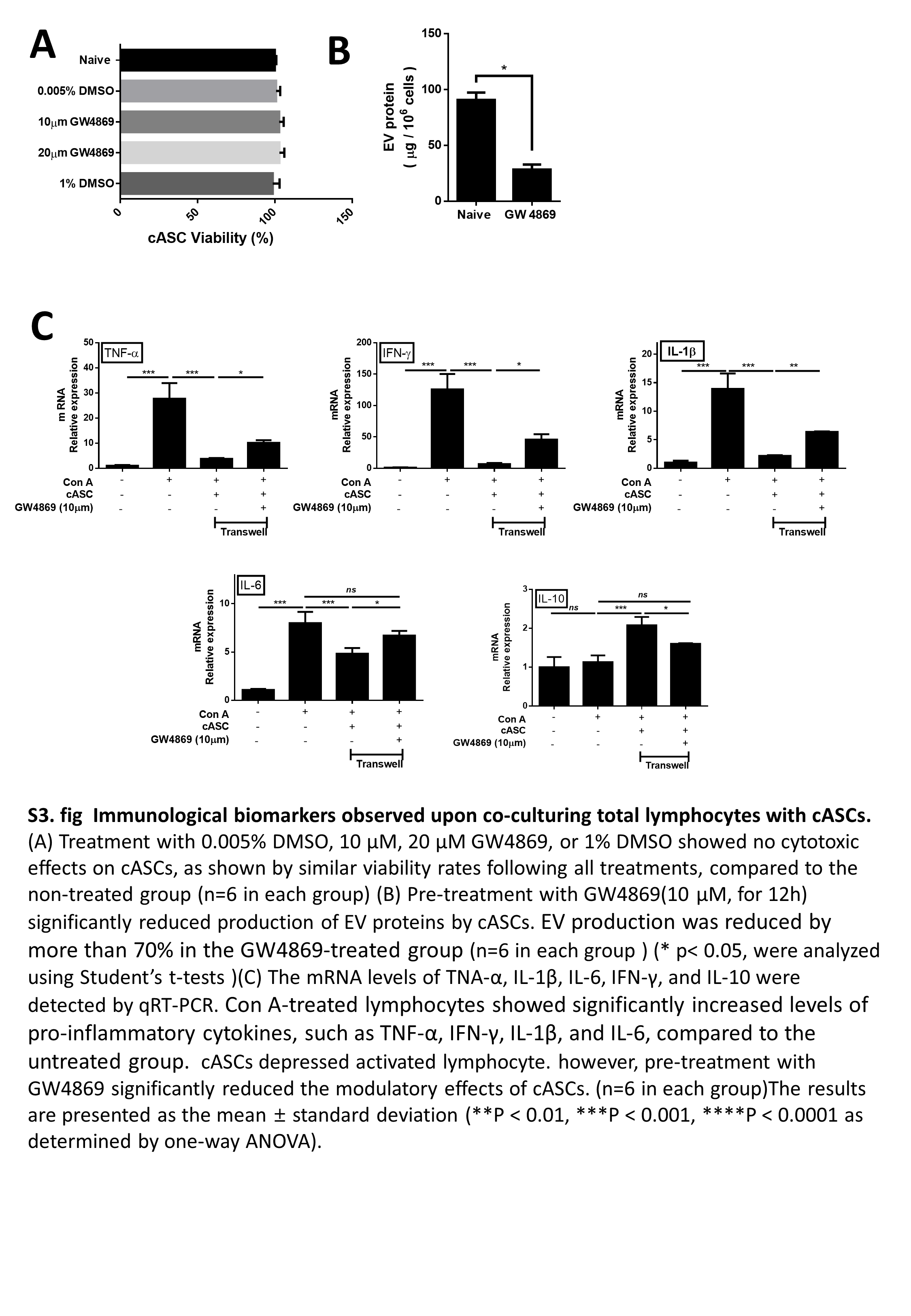

Supplement: S3 Fig — (A) Treatment with 0.005% DMSO, 10 μM, 20 μM GW4869, or 1% DMSO showed no cytotoxic effects on cASCs, as shown by similar viability rates following all treatments, compared to the non-treated group (n = 6 in each group) (B) Pre-treatment with GW4869(10 μM, for 12h) significantly reduced production of EV proteins by cASCs. EV production was reduced by more than 70% in the GW4869-treated group (n = 6 in each group ) (* p< 0.05, were analyzed using Student’s t-tests)(C) The mRNA levels of TNA-α, IL-1β, IL-6, IFN-γ, and IL-10 were detected by qRT-PCR. Con A-treated lymphocytes showed significantly increased levels of pro-inflammatory cytokines, such as TNF-α, IFN-γ, IL-1β, and IL-6, compared to the untreated group. cASCs depressed activated lymphocyte. however, pre-treatment with GW4869 significantly reduced the modulatory effects of cASCs. (n = 6 in each group)The results are presented as the mean ± standard deviation (**P < 0.01, ***P < 0.001, ****P < 0.0001 as determined by one-way ANOVA). (TIF) [file pone.0220756.s003.TIF]

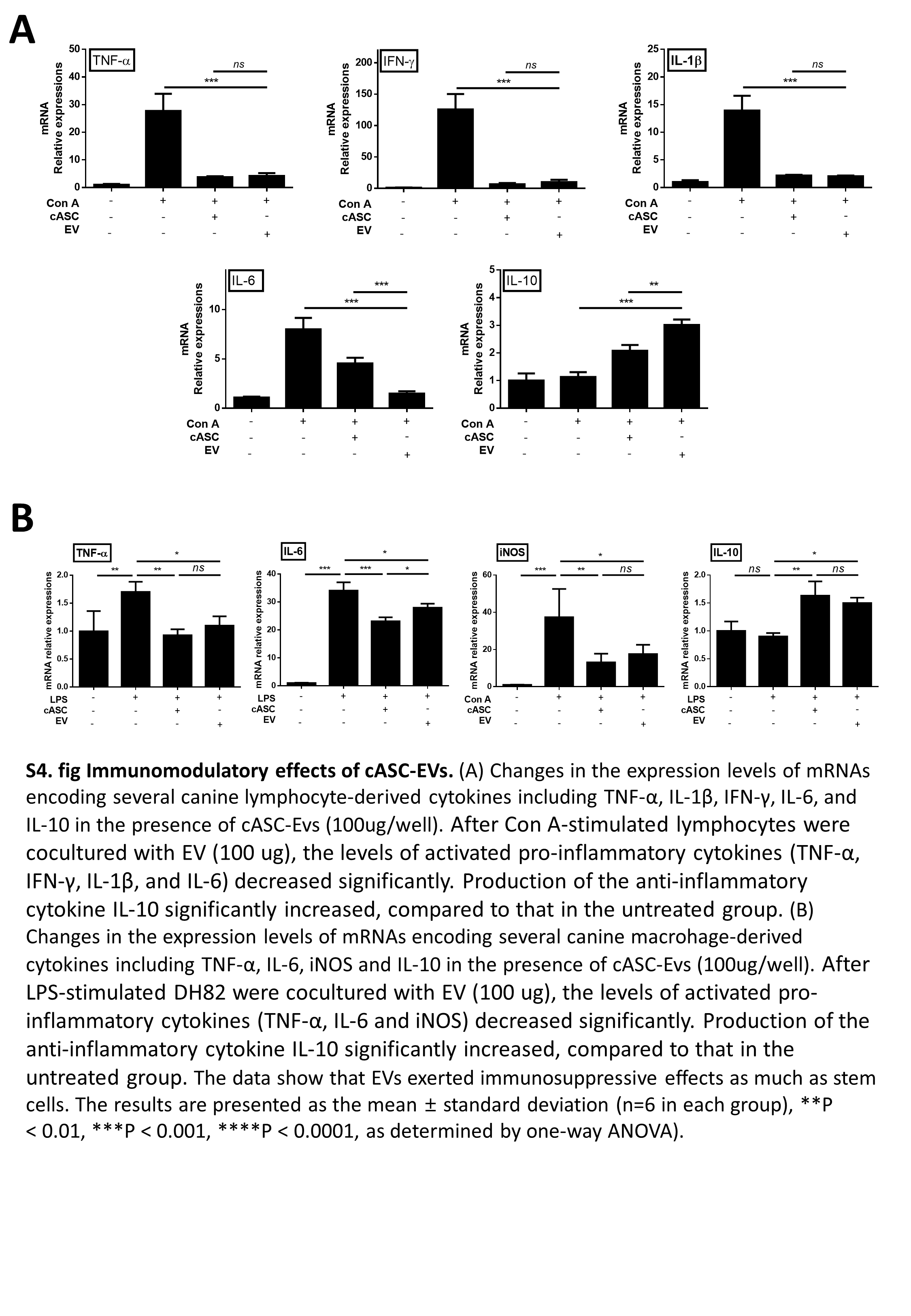

Supplement: S4 Fig — (A) Changes in the expression levels of mRNAs encoding several canine lymphocyte-derived cytokines including TNF-α, IL-1β, IFN-γ, IL-6, and IL-10 in the presence of cASC-Evs (100ug/well). After Con A-stimulated lymphocytes were cocultured with EV (100 ug), the levels of activated pro-inflammatory cytokines (TNF-α, IFN-γ, IL-1β, and IL-6) decreased significantly. Production of the anti-inflammatory cytokine IL-10 significantly increased, compared to that in the untreated group. (B) Changes in the expression levels of mRNAs encoding several canine macrohage-derived cytokines including TNF-α, IL-6, iNOS and IL-10 in the presence of cASC-Evs (100ug/well). After LPS-stimulated DH82 were cocultured with EV (100 ug), the levels of activated pro-inflammatory cytokines (TNF-α, IL-6 and iNOS) decreased significantly. Production of the anti-inflammatory cytokine IL-10 significantly increased, compared to that in the untreated group. The data show that EVs exerted immunosuppressive effects as much as stem cells. The results are presented as the mean ± standard deviation (n = 6 in each group), **P < 0.01, ***P < 0.001, ****P < 0.0001, as determined by one-way ANOVA). (TIF) [file pone.0220756.s004.TIF]

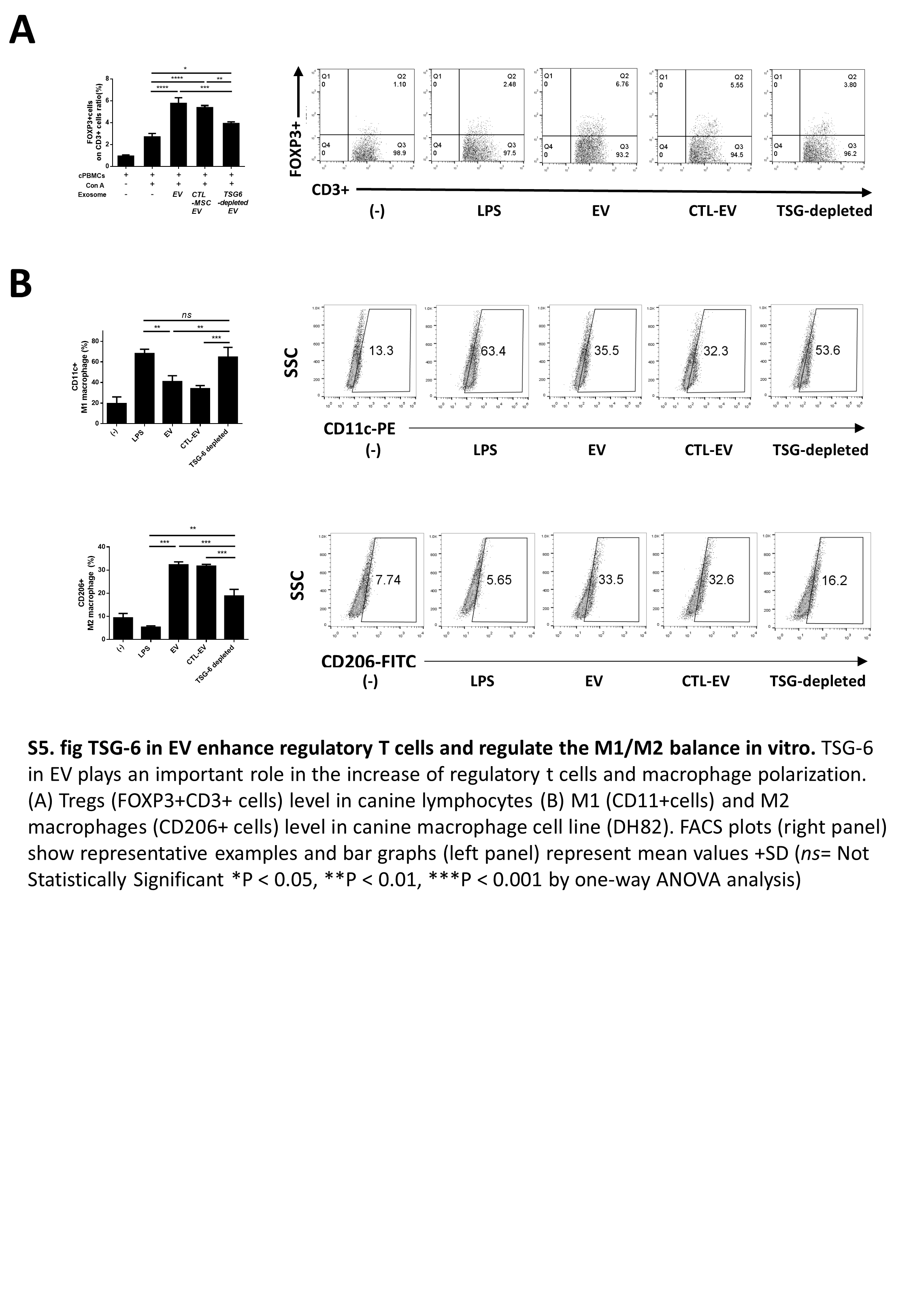

Supplement: S5 Fig — TSG-6 in EV plays an important role in the increase of regulatory t cells and macrophage polarization. (A) Tregs (FOXP3+CD3+ cells) level in canine lymphocytes (B) M1 (CD11+cells) and M2 macrophages (CD206+ cells) level in canine macrophage cell line (DH82). FACS plots (right panel) show representative examples and bar graphs (left panel) represent mean values +SD (ns = Not Statistically Significant *P < 0.05, **P < 0.01, ***P < 0.001 by one-way ANOVA analysis) (TIF) [file pone.0220756.s005.TIF]

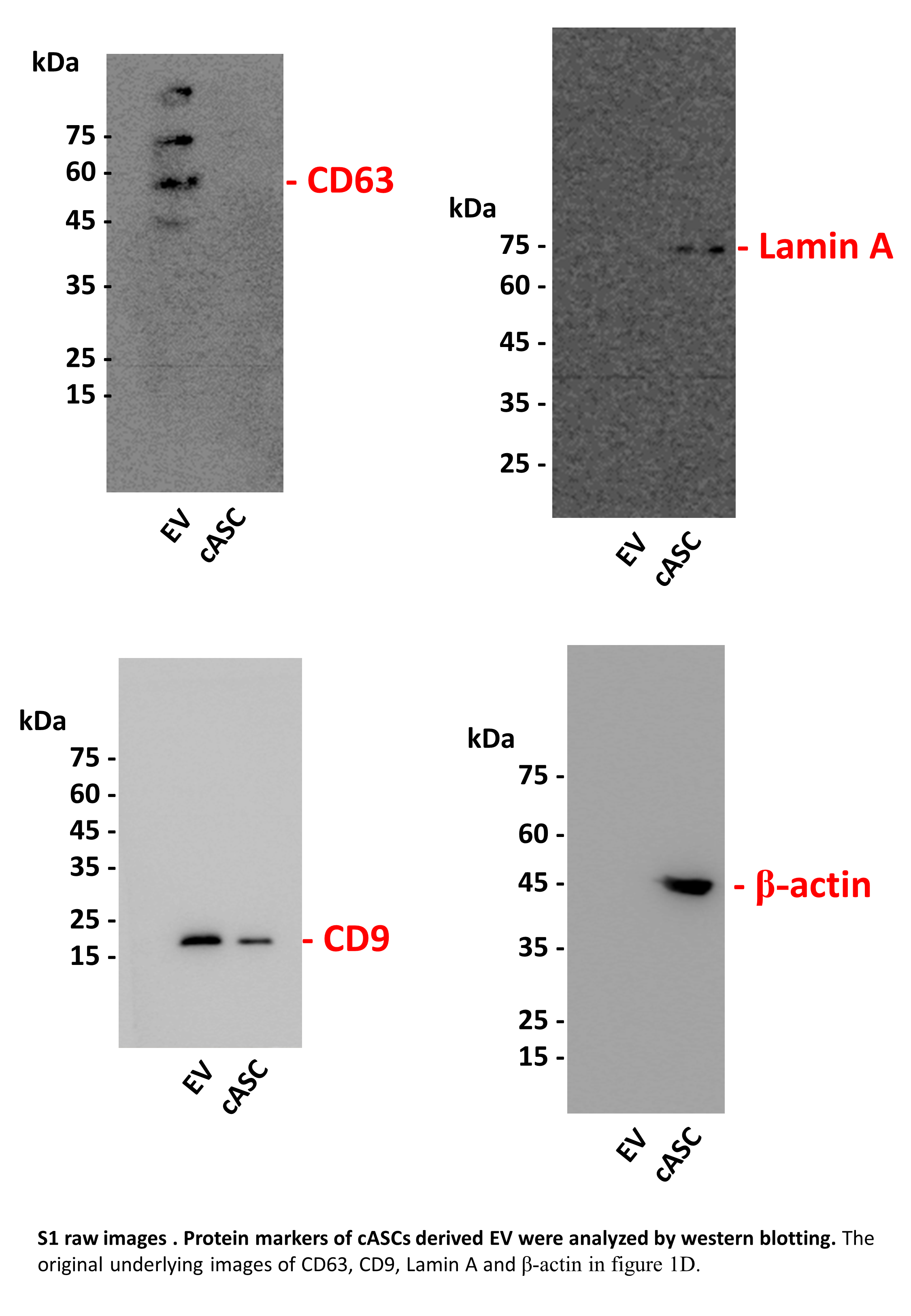

Supplement: S1 Raw Images — The original underlying images of CD63, CD9, Lamin A and β-actin in Fig 1D. (TIF) [file pone.0220756.s006.TIF]
