# Supplementary material for: TSG-6 in extracellular vesicles from canine mesenchymal stem/stromal is a major factor in relieving DSS-induced colitis
Source: PLoS One. 2020 Feb 10;15(2):e0220756. doi: 10.1371/journal.pone.0220756 (PMC7010233; doi:10.1371/journal.pone.0220756)
Supplement: S1 File — (DOCX) [file pone.0220756.s007.docx]

**Additional file 1. Isolation, Culture and Characterization of Canine Adipose Tissue-Derived MSCs**

Adipose tissue (AT) was obtained from a healthy adult female dog during ovariohysterectomy at the Seoul National University Veterinary Medicine Teaching Hospital with the consent of the owner. Procedures were approved by the Institutional Animal Care and Use Committee (IACUC) of Seoul National University (SNU; protocol no. SNU-180502-4). The donor dog was determined not to have any infectious diseases and showed normal blood analysis and imaging findings. Tissue samples were washed 4 times in Dulbecco’s phosphate buffered saline (DPBS; PAN-Biotech, Aidenbach, Germany) with 1% penicillin-streptomycin (PS; PAN-Biotech). Mechanical dissociation was performed with a sterile scissors. Adipose tissue was incubated for 1 h at 37°C in 5% CO_2_ with 0.1% collagenase type 1A (1mg/mL; Sigma-Aldrich) solution. After incubation, same volumes of high glucose Dulbecco’s modified Eagle’s medium (DMEM) with 10% fetal bovine serum (FBS; Pan-Biotech) was added to neutralize the sample then centrifuged at 1200 × g for 10 min. the supernatant was removed, and remaining pellets were suspended with DMEM containing 10% FBS and 1% PS. The cell suspension was passed through a 70 μm cell strainer (Fisher Scientific), then was centrifuged at 1200 × g for 10 min and remove the supernatant. Remaining pellets were resuspended in RBC lysis buffer (Sigma-Aldrich, St. Louis, MO, USA), and incubated at Room temperature for 5 min. then pellets were resuspended with DPBS and centrifugated at 1200 × g for 5 min. the supernatant was removed. Then pellets were resuspended in DMEM containing 10% FBS and 1% PS and transferred to 100mm dishes at a density of 3,000/cm^2^. Cell were incubated at 37 °C and 5% CO_2_ in DMEM with 10% FBS and 1% PS, and medium was replaced every 2 days until the adhered cells showed a fibroblast-like morphology.

The differentiation capacity of the cells was confirmed by identifying differentiated cells using special differentiation media (StemPro Adipogenesis Differentiation, Stem Pro Osteogenesis Differentiation and StemPro Chondrogenesis Differentiation kits; Gibco/Life Technologies) according to the manufacturer’s instruction. Cells in adipogenic and osteogenic differentiation media were each cultured for two weeks, and cells in chondrogenic differentiation medium were cultured for three weeks. After differentiation, all cells were fixed with 4% paraformaldehyde. Cells were then stained with oil red O, 1% alizarin red or alcian blue (all from Sigma-Aldrich) for confirmation of adipocyte, osteoblast, and chondrocyte differentiation, respectively.

In additions, MSCs were tested by cytometry using specific surface marker; being negative for CD34 (FITC), CD45 (FITC), and positive for CD44(FITC), CD90(PE), CD29 (FITC), CD73 (PE) (all from BD Biosciences, Franklin Lakes, NJ, USA). Characterization results were analyzed using Flowjo 7.6.5 software (Tree Star, Inc., Ashland, OR, USA).
